# Deep Learning for the Differential Diagnosis between Transient Osteoporosis and Avascular Necrosis of the Hip

**DOI:** 10.3390/diagnostics12081870

**Published:** 2022-08-02

**Authors:** Michail E. Klontzas, Ioannis Stathis, Konstantinos Spanakis, Aristeidis H. Zibis, Kostas Marias, Apostolos H. Karantanas

**Affiliations:** 1 Department of Medical Imaging, University Hospital, 71110 Heraklion, Greece or miklontzas@gmail.com (M.E.K.); stathisjohn@yahoo.gr (I.S.); vispan@windowslive.com (K.S.); 2 Computational BioMedicine Laboratory, Institute of Computer Science, Foundation for Research and Technology (FORTH), 70013 Heraklion, Greece; kmarias@hmu.gr; 3 Advanced Hybrid Imaging Systems, Institute of Computer Science, Foundation for Research and Technology (FORTH), 70013 Heraklion, Greece; 4 Department of Radiology, School of Medicine, University of Crete, Voutes Campus, 71003 Heraklion, Greece; 5 Department of Anatomy, Medical School, University of Thessaly, 41334 Larissa, Greece; ahzibis@gmail.com; 6 Department of Electrical & Computer Engineering, Hellenic Mediterranean University, 71004 Heraklion, Greece

**Keywords:** hip, avascular necrosis, osteoporosis/transient, deep learning, Artificial Intelligence, InceptionV3, Inception-ResNetV2, VGG-16, transfer learning, MR imaging

## Abstract

Differential diagnosis between avascular necrosis (AVN) and transient osteoporosis of the hip (TOH) can be complicated even for experienced MSK radiologists. Our study attempted to use MR images in order to develop a deep learning methodology with the use of transfer learning and a convolutional neural network (CNN) ensemble, for the accurate differentiation between the two diseases. An augmented dataset of 210 hips with TOH and 210 hips with AVN was used to finetune three ImageNet-trained CNNs (VGG-16, InceptionResNetV2, and InceptionV3). An ensemble decision was reached in a hard-voting manner by selecting the outcome voted by at least two of the CNNs. Inception-ResNet-V2 achieved the highest AUC (97.62%) similar to the model ensemble, followed by InceptionV3 (AUC of 96.82%) and VGG-16 (AUC 96.03%). Precision for the diagnosis of AVN and recall for the detection of TOH were higher in the model ensemble compared to Inception-ResNet-V2. Ensemble performance was significantly higher than that of an MSK radiologist and a fellow (*P* < 0.001). Deep learning was highly successful in distinguishing TOH from AVN, with a potential to aid treatment decisions and lead to the avoidance of unnecessary surgery.

## 1. Introduction

Differential diagnosis between transient osteoporosis (TOH) and avascular necrosis of the hip (AVN) presents a significant diagnostic challenge [1]. A variety of methods have been proposed to accurately differentiate between the two entities, increasing the diagnostic accuracy of radiologists and allowing non-musculoskeletal (MSK) radiologists to differentiate between the two entities. Presence of the “sparing” sign has been shown to be characteristic in 87.7% of TOH cases [2], whereas bone marrow edema (BME) is present only in later stages of AVN. In addition, subchondral fractures of TOH are not similar in appearance to the subchondral “band-like” or the “crescent” sign encountered in AVN [3,4]. No pathophysiological similarity has been found between the two entities [5], and it has been demonstrated that patients with TOH do not progress to AVN, as previously thought [2,6]. Our group previously developed a radiomics–machine learning pipeline that allows differential diagnosis with an area under the curve (AUC) that is equal to MSK radiologists’ and better than general radiologists’ [7]. Accurate differentiation between the two entities is of the utmost importance, since it determines whether the patient will undergo conservative treatment for the management of TOH or surgical treatment (core decompression, vascularized fibular grafting, etc.) for the management of AVN [8]. Correct diagnosis even in non-experienced MRI readers may avoid unnecessary surgery, with a huge benefit for the patient.

Radiomics analysis such as the one presented in our previous work [7] has been widely used for the analysis of features derived from medical imaging [9]. Traditional radiomics include the segmentation of a region of interest, the extraction of radiomics features using established algorithms, and the use of machine learning techniques for the development of classifiers that allow accurate diagnosis of a disease [10,11]. This pipeline allows the detailed evaluation of imaging features and group classification with the use of machine learning algorithms [12]. High-throughput extraction of radiomics features allows for detailed characterization of medical images and the development of high-fidelity machine learning models [12,13]. However, such strategies also require significant computer expertise of the users and include steps of manual or automatic segmentation and data curation that increase the complexity of the method. Deep learning overcomes the need for segmentation and manual data extraction, automating a process encountered by radiologists in everyday clinical practice.

The aim of the current manuscript is to develop a deep learning strategy that allows fully automated differentiation between TOH and AVN, reducing the need for manual analysis related to radiomics. Transfer learning was used to train a series of deep learning architectures, which were subsequently fine-tuned with the use of MR images. A model ensemble was also developed, which provided a consensus decision between three deep learning models in an attempt to increase decision accuracy. Ultimately, the performance of the developed deep learning strategies was compared to the diagnostic performance of an MSK radiologist and an MSK imaging fellow.

## 2. Materials and Methods

### 2.1. Patients

The multi-institutional patient cohort utilized in this work was previously used for the development of radiomics signatures of TOH and AVN [7]. Study collection for dataset development was performed in an MSK radiology department specializing in bone marrow imaging and receiving domestic and international referrals for second opinions on complicated AVN and TOH cases. Briefly, 109 consecutive hips with TOH (107 patients) and 104 consecutive hips with AVN (67 patients) were retrospectively included in this study between July 2014 and March 2020. All patients were examined in the specialized bone marrow imaging clinic of our University Hospital. Ground truth diagnosis was established with a pipeline utilizing clinical and imaging data, and all patients had a follow-up of at least 1 year to establish the spontaneous resolution of TOH or the persistence of AVN. Patients with disorders that can manifest with BME of the proximal femur or insufficient follow-up (<1 year) to establish the diagnosis (AVN vs. TOH) were excluded, as previously described (prior trauma, infection, inflammatory arthropathies, tumors, and prior surgery). Institutional review board approval was received (Ref. No. 360/08/29-04-2020), all patients provided consent to undergo the examination, and the study was performed according to the Declaration of Helsinki, with a waiver of consent for the use of anonymized retrospective data.

### 2.2. MR Imaging

MRI examinations were performed at multiple 1.5 or 3T MRI machines and included (a) coronal T1-w, (b) coronal Short Tau Inversion Recovery (STIR) sequences, (c) axial fat suppressed PD/T2-w, and (d) high-resolution 3D gradient echo sequences of the affected hip. In the case that the initial examinations did not contain the required sequences or suffered from low image quality, the examination was repeated in our institution using a 1.5T machine (Vision/Sonata, Siemens, Erlangen), and the revised images were used instead. Imaging examinations were evaluated by an MSK radiologist with 35 years of experience, and ground truth was established by combining clinical and MRI data to determine the progression of disease at follow-up. Specifically, the diagnosis of AVN was established when the band-like sign was identified on T1-w images along with the single line sign on STIR or fat suppressed PD/T2-w sequences [14]. It should be noted that the “double-line” sign, which was used in the past for the diagnosis of AVN, was not used since it has been established that it represents a chemical shift artifact [15]. In advanced stages of the disease (ARCO ≥ 3), a subchondral fracture can be identified in fluid-sensitive sequences [16]. The diagnosis of TOH was established in patients with acute onset of symptoms and the appearance of extensive bone marrow edema of the proximal femur with or without the “sparing sign” and with or without low signal intensity lines representing subchondral fractures, which can be found in almost 50% of TOH patients deep within the subchondral marrow [2]. In order to confirm the diagnosis of TOH for the purposes of establishing a robust ground truth, resolution of the edema was confirmed in follow-up examination (≥1 year). This is characteristic of TOH, which resolves only with the use of conservative measures (reduced weightbearing), in contrast to AVN, which is a progressive disease that does not resolve only with weightbearing reduction. In clinical practice, there is currently no other way to establish the diagnosis with confidence. All diagnostic decisions were discussed and reached in consensus with the referring surgeon. The MRI features used for the diagnosis of AVN and TOH have been described in detail previously [7]. Deep learning models were trained and validated with the use of mid-coronal STIR images through the femoral head and neck, since the pattern of BME on STIR images is sufficient for the imaging diagnosis of the two conditions in everyday MSK radiology practice; STIR is the sequence of choice for the depiction of bone marrow edema, which is a key feature of both conditions.

### 2.3. Data Preparation, Deep Learning, and Comparison to Experts

The study was performed according to the Checklist for Artificial Intelligence in Medical Imaging (CLAIM) guidelines [17]. Curation of the dataset prior to model development and training included splitting into training and validation cohorts, with a ratio of 70:30, and images of each group were processed using grey level harmonization and histogram normalization to reduce MR scanner-related variability across our dataset while avoiding data leakage [18,19]. The testing dataset served as the external validation set, since the MRI examinations were collected from multiple scanners at different centers, ensuring that our models were not center-specific. Prior to use in deep learning training, all images were resized to 150 × 150 px, and the dataset was augmented by clockwise/anti-clockwise rotation (10°) and horizontal image flipping to reach a total of 420 images (210 TOH and 210 AVN). This achieves a reduction of bias related to group imbalance and model exposure to a bigger dataset.

**Figure 1:**
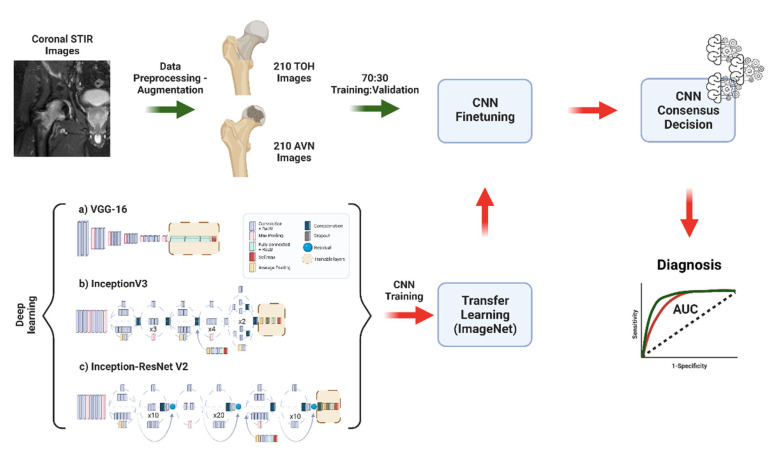
Flow diagram describing methodology followed for data augmentation, deep learning model training with transfer learning, and the development of a model ensemble for the diagnosis of TOH vs. AVN (created with BioRender.com).

A transfer learning strategy was utilized as previously published [20,21]. Briefly, three convolutional neural network (CNN) architectures were utilized—InceptionV3, VGG-16, and Inception-ResNetV2—and their initial weights were acquired using transfer learning from the ImageNet dataset [22]. All three CNN architectures have been developed to handle image recognition applications and have been widely used in image-based applications with excellent results. This allows the networks to take advantage of the large ImageNet dataset, which includes more than 14 million images of several classes. Performing the initial training of the networks using this dataset allows CNNs to use existing knowledge present in a huge dataset, enabling them to identify features that cannot be learned by a limited disease dataset [23]. Networks were subsequently finetuned by training in 70% of our data and validation in 30% of the data. CNN training was performed for a maximum of 50 epochs utilizing an early stopping function. Overfitting was also avoided by visual assessment of training/validation accuracy and training/validation loss plots. In cases where training loss continued to decrease at high epoch values while loss in the validation set started to increase, overfitting was considered. This was avoided in our work with the use of the early stopping function. To develop the ensemble decision of the models, a hard-voting strategy was adopted, where a consensus decision was reached when at least two of the three CNNs agreed, and this was recorded as the decision of the ensemble. Models were developed in Python v3.8 in Jupyter Notebook v6.4 and were trained and validated using an NVIDIA GeForce RTX 2060 Super 8 GB GPU in a 64 bit Windows-based workstation with 32 GB RAM and Intel Core i7-10700F CPU at 2.9 GHz. Model development was performed with the Keras framework and the Tensorflow backend. The methodology used for model development and training is summarized in Figure 1.

CNN performance was compared to the performance of one MSK radiologist with 7 years of experience and a senior MSK imaging fellow. Both readers have worked in a center with an expertise on bone marrow imaging and were asked to blindly diagnose the images of the training dataset, assigning them to the AVN or TOH group.

### 2.4. Statistical Analysis

Model performance was assessed by means of precision, recall, and f1-score. Sensitivity, specificity, positive predictive value (PPV), and negative predictive value (NPV) were also provided for CNNs and radiologists. Reader and CNN performance comparison was achieved with the use of Receiver Operating Characteristics (ROCs). For each curve, bootstrapping was used to calculate the Area Under the Curve (AUC) and the respective 95% confidence interval. Significance between CNN and reader performance was evaluated using DeLong′s method [24], and significance was considered with a *P*-value lower than a = 0.05. ROC curve analysis was performed with R (v. 4.03, https://www.R-project.org/ (accessed on 26 July 2022)).

## 3. Results

### 3.1. Deep Learning Model Training with Transfer Learning

**Figure 2:**
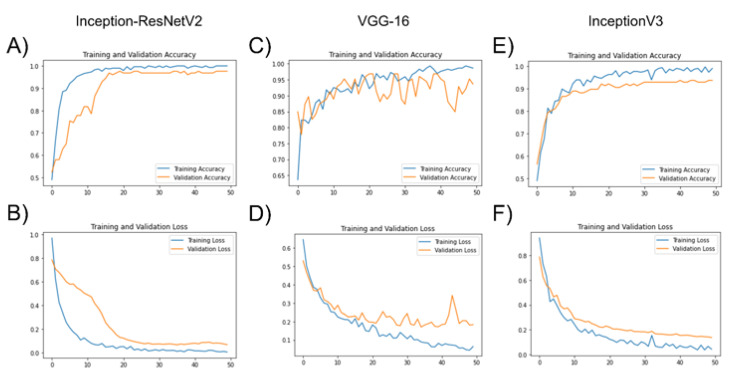
Training/validation accuracy (**A**,**C**,**E**) and training/validation loss (**B**,**D**,**F**) plots for the finetuning for the ImageNet pretrained Inception-ResNetV2 (**A**,**B**), VGG-16 (**C**,**D**), and Inception V3 (**E**,**F**).

Deep learning model training was performed without any evidence of overfitting, achieved by the use of early stopping and verified by the visual inspection of performance (training/validation accuracy) and optimization (training/validation loss plots) learning curves (Figure 2). Validation loss decreased and showed no increased trend towards higher epochs for all model architectures, indicating lack of overfitting.

**Figure 3:**
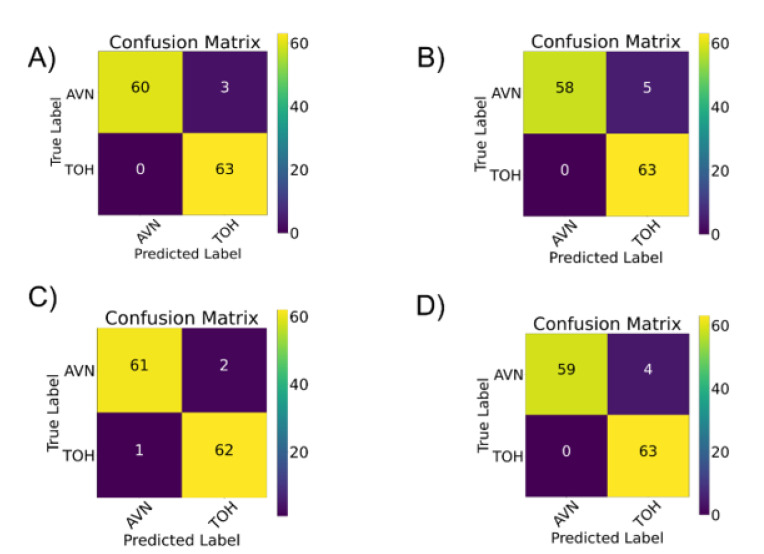
Confusion matrices for the ensemble CNN decision (**A**), for VGG-16 (**B**), InceptionV3 (**C**) and Inception-ResNetV2 (**D**). TOH: transient osteoporosis of the hip; AVN: avascular necrosis of the femoral head.

Diagnostic performance of each individual CNN and the model ensemble was found to range (AUC) between 96.03% and 97.62% (Table 1). Interestingly, InceptionResNet-V2 achieved the same total AUC compared to the model ensemble. However, the ensemble reached a perfect (100%) precision score for AVN and recall score for TOH, with a 0% type I error for the diagnosis of TOH (Table 1 and Figure 3). As demonstrated in Figure 3, all CNNs had more AVNs confused for TOH (type I error) with the ensemble, VGG-16, InceptionV3 and Inception-ResNetV2 counting 3, 5, 2 and 4 AVNs predicted as TOH, respectively. Nonetheless almost all TOH cases were correctly predicted as TOH, with 0 false negatives for the ensemble, VGG-16 and Inception-ResNetV2 and only one false negative case (type II error) predicted by InceptionV3.

### 3.2. Comparison of Deep Learning to Expert Readers

**Figure 4:**
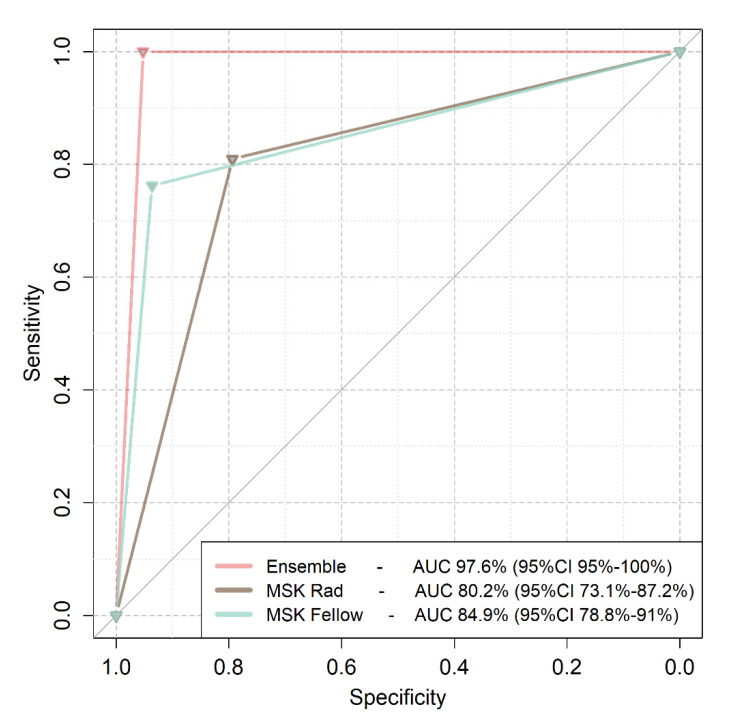
Receiver Operating Characteristics (ROC) curves of the model ensemble and MSK imaging experts. Model ensemble curve is plotted as a pink line, the MSK radiologist curve is plotted with brown color and the MSK fellow curve is plotted with a turquoise color. MSK Rad: Musculoskeletal Radiologist; AUC: Area Under the Curve.

Performance of the model ensemble was compared to the performance of expert readers in diagnosing the images of the validation dataset. Both readers were sufficiently trained in MSK radiology at a dedicated department specializing in bone marrow imaging, which receives referrals for second opinions on complicated AVN and TOH cases. The model ensemble achieved an AUC of 97.6% (95%CI from 95% to 100%), the MSK radiologist an AUC of 80.2% (95%CI from 73.1 to 87.2%) and the fellow an AUC of 84.9% (95%CI from 78.8% to 91%). Ensemble performance was better than both experts (*p* < 0.001). No statistically significant difference was found between the performance of the MSK radiologist and the MSK fellow (*p* = 0.23) (Figure 4).

## 4. Discussion

Herein, we present a deep learning methodology to differentiate between TOH and AVN of the hip. Three CNN architectures were trained with a transfer learning methodology, and their performance in diagnosing between the two diseases was evaluated in a multi-institutional dataset. Importantly, the performance of the model was found to be significantly higher that two experts in MSK radiology. The results presented in this manuscript set the basis for the automation of this diagnostic process, which requires significant expertise in MSK imaging. Accurate model predictions can assist the diagnostic process while preventing unnecessary surgery in cases of misdiagnosed AVN.

Differentiation between TOH and AVN has been previously attempted by means of traditional imaging and by means of machine learning. Imaging features characteristic of TOH include the “sparing sign” and extensive edema, in combination with a history of acute onset of pain in the absence of trauma [2,5,25,26,27]. Fractures can be found in both conditions, but the fractures of TOH with a deep subchondral thin and irregular line do not resemble the articular collapse fractures of AVN. The diagnosis of AVN can be made with the use of the “band-like” and the “crescent” sign [1]. Another important feature helpful in traditional diagnosis is that BME characterizes later stages of AVN (ARCO > 3). In an attempt to solve the diagnostic dilemma, Geith et al. used perfusion patterns in dynamic contrast enhanced MRI [28]. However, their attempt suffered from a low sample size (*n* = 32), which does not allow generalization of the method. Our group previously developed a radiomics pipeline that achieves an AUC ≅ 94%, which was superior or equal to radiologist diagnoses [7]. The methodology presented herein offers superior performance (AUC ≅ 98%) while automating the analysis, which in radiomics is largely manual and requires expertise in data analysis, while avoiding the bias related to segmentation of the region of interest at the proximal femur [29]. Similar CNN ensemble methodology has been previously used in the differential diagnosis of BME in the knee with excellent predictions, better or similar to MSK experts [20].

Ensemble performance was superior to both expert readers. This was not the case when radiomics was used to discriminate between the two entities [7]. Radiomics-based machine learning achieved a performance equal to MSK radiologists and fellows and higher than a general radiologist. This can be attributed to the higher diagnostic performance of the deep learning ensemble, which outperformed the radiomics-based model. Interestingly, the diagnostic performance of the readers in this work ranged between 80.2% and 84.9%, whereas such performance was previously found to range between 87.2% and 90.6% [7]. This discrepancy can be attributed to differences in reader experience as well as to augmentation-related differences in the final testing dataset used in the present study. In our study, both readers have been trained in a center with a dedicated bone marrow imaging service and extensive experience in both TOH and AVN. In addition, individual CNNs achieved a performance similar to the performance of the ensemble. However, the ensemble achieved the maximum precision and recall and the minimum type I and type II error, which is of utmost importance in diagnostic decisions that will determine the need for a surgical operation. Therefore, solutions with the minimum false negative and false positive rates should be preferred over others with similar total AUC.

Performance of the CNN ensemble and each individual network was comparable to the published literature. Voting ensembles have been used by Tasci et al. for the diagnosis of tuberculosis on plain radiographs using a combination of Inception and Xception models with several data preprocessing/augmentation steps [30]. Their results demonstrate an accuracy of approximately 97% for the diagnosis of the disease. Tsiknakis et al. [21] used a similar ensemble to classify an image dataset with 20 categories. The authors used a voting ensemble strategy and a combination of InceptionV3, Xception, ResNet and Inception–ResNet to classify images to twenty categories, achieving an AUC close to 100%. Similar results have been previously demonstrated by our group for the differentiation between subchondral insufficiency fractures and advanced osteoarthritis of the knee [20] using the same CNN architectures and a hard voting ensemble. The results presented herein demonstrate a higher overall performance of the ensemble, which was accompanied by a higher performance of each one of the individual CNNs. Higher performance of individual CNNs suggests that they may also be used independently without the use of voting. However, voting was found to offer higher precision and recall, which are important for medical diagnosis, especially when the decision may lead to a total hip arthroplasty.

The method presented in this manuscript has an important clinical implication related to the management of patients’ diagnoses with AVN, which might result in surgery. In everyday clinical practice, there are imaging findings that can differentiate both disease entities, especially in the trained eyes of an MSK radiologist. Diagnosis becomes more complicated when AVN is accompanied by a subchondral fracture and at the latest stages of the disease, when the disease is accompanied by bone marrow edema [16,31]. The value of our work lies on the demonstration of a method that achieves high diagnostic performance without the need for expertise in MSK radiology. The addition of such an application to the everyday clinical workflow may assist everyday diagnostic decisions, providing an additional tool for diagnostic consulting purposes. Ultimately, being a python-based application, it can be incorporated into python-friendly RIS-PACS systems, allowing for seamless integration into the everyday diagnostic workflow [32]. The capability of PACS incorporation is extremely important according to the FUTURE-AI guidelines, since it increases the usability of the algorithm [32].

Our study has specific strengths, such as the sample size, which is large given the low prevalence of both diseases. In addition, the CNNs presented in this study were trained with the use of multi-vendor images, ensuring the generalizability of our results to MR images obtained from different centers. It has been demonstrated that between-scanner batch effect is more important than same scanner variability, and it is currently widely accepted that sufficient training of deep learning models should include images acquired in multiple MRI machines in order to ensure generalizability [33]. The comparison of our results to the diagnosis of MSK radiologists at different levels (expert, fellow) is another advantage of our strategy. Limitations of our study include the retrospective collection of data (which is mandatory given the low prevalence of the disease and the need for large datasets for deep learning). Finally, using only STIR images for the diagnosis could be considered a limitation of our study. Nonetheless, STIR images are sufficient for the diagnosis of both conditions in everyday radiological practice because they are the gold standard for the evaluation of BME.

## 5. Conclusions

In conclusion, our work proposes an automated methodology utilizing CNNs and transfer learning for the accurate differentiation between TOH and AVN. The suggested pipeline leads to a diagnostic performance that is higher than expert readers. The use of such an automated tool has the potential to revolutionize the differential diagnosis between the two entities, contributing to correct diagnostic decisions and avoiding unnecessary surgery.

## Figures and Tables

**Table 1:** Performance metrics of individual convolutional neural networks and the respective network ensemble.

	AUC	Group	Precision	Recall	f1-Score
**Model ensemble**	**97.62%**				
		AVN	1	0.95	0.98
		TOH	0.95	1	0.98
**VGG-16**	**96.03%**				
		AVN	1	0.92	0.96
		TOH	0.93	1	0.96
**InceptionV3**	**96.82%**				
		AVN	1	0.94	0.97
		TOH	0.94	1	0.97
**Inception-ResNet-V2**	**97.62%**				
		AVN	0.98	0.97	0.98
		TOH	0.97	0.98	0.98

AUC: Area Under the Curve; AVN: Avascular Necrosis; TOH: Transient Osteoporosis of the Hip.

## Data Availability

Data not contained in the manuscript are available from the corresponding author upon reasonable request.
